# Epidemiología del autocuidado, más allá de lo individual y lo sanitario

**DOI:** 10.7705/biomedica.5761

**Published:** 2020-11-12

**Authors:** José Moreno-Montoya

**Affiliations:** 1 Subdirección de Estudios Clínicos, Fundación Santa Fe de Bogotá, Bogotá, D.C., Colombia Subdirección de Estudios Clínicos Fundación Santa Fe de Bogotá BogotáD.C Colombia

**Keywords:** infecciones por coronavirus, síndrome respiratorio agudo grave, virus del SRAS, autocuidado, vacunas, epidemiología, salud pública, Coronavirus infections, severe acute respiratory síndrome, SARS virus, self-care, vaccines, epidemiology, public health

## Abstract

En medio de la crisis pandémica a nivel global, la preocupación internacional ha girado en torno a la adopción de medidas de control y prevención orientadas a la reducción de la velocidad de propagación del virus en espera de que se disponga de una medida sanitaria radical como la vacuna. El esfuerzo gubernamental y social ha tenido un gran impacto en diversos sectores de la sociedad y las consecuencias han superado el ámbito sanitario. En este ensayo se discute su alcance en el sentido de la apropiación de las medidas de control del riesgo y se propone el método epidemiológico como una alternativa que va más allá de la cuantificación de los riesgos y la atribución de responsabilidades. Por último, se plantea la necesidad de fomentar procesos de socialización de la información que ayuden a la comprensión de las consecuencias de los actos individuales y favorezcan la superación de la expectativa de control pandémico únicamente basada en el uso de medidas coercitivas.

## Abstract (en)

Amid the global pandemic crisis, international concern has centered on the control and prevention measures aimed at reducing the speed of the virus transmission while a more radical sanitary measure, such as vaccines, is achieved. Governmental and social efforts have had great impact on various sectors of society and their consequences have exceeded the sphere of health.

This essay discusses the scope of specific measures in the sense of the appropriation of risk control measures and proposes the epidemiological method as an alternative that goes beyond the quantification of risks and the attribution of responsibilities. To conclude, the emphasis is placed on the need to promote information about socialization processes to better understand the consequences of individual acts favoring alternatives other than pandemic control based on the use of coercive measures.

Diversas organizaciones a nivel mundial han promovido el desarrollo de medidas cuya finalidad principal es la desaceleración del contagio masivo de lo que, hasta ahora, parece no tener solución inmediata: la pandemia de COVID-19, cuyas consecuencias superan por mucho el problema de la mortalidad y la morbilidad ^1^. En este sentido, buena parte de los esfuerzos científicos se han centrado hasta ahora en el desarrollo de vacunas aptas para su aplicación masiva en la población. Como es natural, la complejidad de los procesos técnicos y los aspectos éticos de esta tarea entraña desafíos: se ha estimado que el desarrollo exitoso de una vacuna puede tomar, por lo menos, 18 meses, tiempo crucial para caracterizar el posible antígeno y el modelo animal, así como la efectividad y la seguridad esperadas de cualquiera de las vacunas candidatas ^2^.

En este contexto, mientras se logra el desarrollo de vacunas óptimas y se masifica su producción y distribución, los entes gubernamentales y sanitarios alrededor del mundo han implementado diversas medidas interinas que buscan disminuir la velocidad de la transmisión y el impacto de la COVID-19 en la sociedad. Tales esfuerzos han incluido medidas de estricta restricción social, como el confinamiento, la ley seca y el cierre escolar, con la consecuente merma de las actividades industriales, comerciales y productivas ^3^. Las preocupaciones derivadas del efecto de dichas iniciativas han superado, naturalmente, la esfera de lo sanitario y afectan de manera no menos grave ámbitos como la sostenibilidad económica, el empleo y la capacidad fiscal de los gobiernos que, en aras de mantener a salvo a sus pueblos y sus sistemas sanitarios, desplazan y postergan inversiones de gran magnitud, con el fin de garantizar los recursos necesarios para el adecuado control de los efectos de la pandemia, lo que inevitablemente afecta otros aspectos de los sistemas de protección y desarrollo social ^4^.

En este proceso, y como parte de la urgente necesidad de información frente a una situación desconocida en el ámbito sanitario mundial del último siglo, es común encontrar en los medios de comunicación información sobre los avances en la compra e instalación de equipos de respiración asistida para pacientes con graves insuficiencias respiratorias derivadas del contagio, así como sobre promisorias alternativas farmacéuticas e, incluso, opciones menos ortodoxas, algunas discutibles, y otras que requieren tiempo dada la rigurosidad científica necesaria en estos casos y la escasez de ensayos clínicos que proporcionen el adecuado sustento a las decisiones médicas ^5^.

Tal masificación mediática ha impulsado el resurgimiento de la epidemiología como componente fundamental de la salud pública y ha generado una avalancha de opiniones que exceden los ámbitos médico y científico e inundan nuestra cotidianidad, planteándonos nuevos desafíos en torno a la necesidad constante de mejorar los procesos de reporte y socialización de la información ^6^. Así las cosas, abundan por estos días los expertos que en profundas disertaciones se esfuerzan por proporcionar claridad sobre los alcances, limitaciones y tiempos requeridos para lograr la anhelada cura. Ha sido tal el alud de información, que las entidades gubernamentales en cada país han debido liderar procesos que velen por la rigurosidad en la divulgación de los datos relativos a la sorpresiva invasión del virus y sus consecuencias ^7^.

Y no es para menos. Pocos esperaban, entre ellos los más versados epidemiólogos, el ataque de un enemigo microscópico que pusiera en entredicho la efectividad de nuestros procesos sanitarios y entrara a saco en las arcas gubernamentales, industriales y personales. En ese contexto, la posibilidad de acceder a una vacuna o un fármaco efectivo ha revelado la profunda brecha de las desigualdades económicas y de acceso entre los países ^8^, pues las economías poderosas ya han empezado a asegurar una adecuada participación en la distribución del producto biológico salvador, en tanto que aquellos países con recursos limitados o mal distribuidos aguardan por una oportunidad de compra.

La situación trasciende el ámbito internacional y se ha hecho evidente en cada país en la lucha diaria de quienes no tienen la opción de una sala cómoda de hospital y se debaten entre el hambre, la urgencia de conseguir el sustento diario y las medidas de prevención que, de un modo u otro, afectan sus débiles economías ^9^. Muchos hogares y grupos enteros de la población deben enfrentar estas preocupaciones además de la amenaza del SARS-Cov2. Su bienestar implica derechos que van más allá de lo sanitario y ello invita a la discusión de aspectos sociales como la posibilidad del teletrabajo, de desplazarse, de educar sus hijos y de disfrutar de su libertad.

Dichas limitaciones sociales han abonado el terreno para el restablecimiento del puesto que la ciencia reclama en la sociedad actual. Ante la urgencia de respuestas, se le ha delegado a esta toda la responsabilidad y ahora recae sobre sus hombros la obligación de una solución que libere a los sujetos individuales de sus propias responsabilidades de autocuidado y garantice el retorno a una normalidad que, nueva o no, permita el libre desarrollo de las actividades que antes ocultaban la verdadera dimensión de problemas a los que solo se les prestaba apenas la atención usual.

A esto debe añadirse el ya conocido horizonte de las inequidades, ante lo cual urge considerar el avance real de nuestras sociedades como colectivos desarrollados y sostenibles, capaces, entre otros deberes, de velar por su propio cuidado ^10^. Las vacunas, los fármacos, las pociones mágicas o cualquier solución que nuestros experimentados científicos logren desarrollar, no remplazan la necesidad de trasladar también a los sujetos la responsabilidad de la atención, porque ellos, justamente por ser los responsables del contagio, son los primeros llamados a su control. Además, el manejo pandémico tampoco da la debida consideración a la simultánea presencia de otras problemáticas tanto o más graves que la COVID-19, cuyas consecuencias son potencialmente catastróficas, como el cambio climático, las guerras y la violencia en sus diversas formas, entre muchas otras ^11^^,^^12^.

Medidas como el lavado de manos, el uso de tapabocas, la limitación de las reuniones sociales y el distanciamiento físico no son suficientes a pesar de su efecto positivo ^12^. Quizá la solución más efectiva está al alcance de nuestras manos, y el propósito de tener una cantidad adecuada de salas de atención médica disponibles y justos niveles de acceso a los servicios sanitarios para así retomar las actividades productivas y académicas y todo aquello que empezamos a extrañar, no dependa de una conjetura química o genética, sino pedagógica y de responsabilidad social ^13^. Con la COVID-19 ha llegado la hora de asumir el desafío de una epidemiología que vaya más allá de lo individual y lo sanitario.

Sin embargo, la responsabilidad de las acciones individuales no depende completamente de los sujetos en sí mismos. En las sociedades organizadas es deber primordial de las instituciones y organizaciones gubernamentales y sociales favorecer la implementación de las medidas necesarias, de largo aliento, que promuevan el desarrollo de las capacidades de individualización en el control del riesgo, por lo menos ante las epidemias, y siembren la semilla necesaria de conciencia del acto individual en favor del bien colectivo ^14^. Huelga decir que las medidas de choque, como los toques de queda, reflejan en el fondo la incapacidad de la voluntad popular en pro del beneficio derivado de limitar sus acciones individuales ^15^.

La pandemia deja un mensaje claro. La crisis no es solo un problema sanitario y su caracterización epidemiológica no puede responder únicamente a una visión exclusivamente biológica y médica sino también de alcances sociales, económicos y culturales de profundo arraigo poblacional ^16^. Si bien las herramientas del método epidemiológico favorecen la cuantificación de los acontecimientos y resultan de utilidad para la validación de las relaciones plausibles entre las variables principales en una crisis sanitaria, en el futuro los esfuerzos deben orientarse a la aplicación de los modelos epidemiológicos como punto de apoyo para la valoración de la salud mediante un enfoque que integre el ámbito económico, las políticas de equidad y la normatividad, pero, sobre todo, la responsabilidad individual y pública. Es el momento de promover y facilitar la socialización de cifras y estimaciones que, en el marco de la epidemiología moderna y sus métodos, ayuden a comprender la gravedad y las consecuencias de las acciones individuales y gubernamentales y a dejar de lado las medidas coercitivas como únicas soluciones ante una crisis como la provocada por la pandemia de COVID-19.
